# Digital Detection of Exosomes by Interferometric Imaging

**DOI:** 10.1038/srep37246

**Published:** 2016-11-17

**Authors:** George G. Daaboul, Paola Gagni, Luisa Benussi, Paolo Bettotti, Miriam Ciani, Marina Cretich, David S. Freedman, Roberta Ghidoni, Ayca Yalcin Ozkumur, Chiara Piotto, Davide Prosperi, Benedetta Santini, M. Selim Ünlü, Marcella Chiari

**Affiliations:** 1 Nexgen Arrays, Boston, Massachusetts 02215, USA; 2 Consiglio Nazionale delle Ricerche, Istituto di Chimica del Riconoscimento Molecolare (ICRM), Milano, Italy; 3 Molecular Markers Laboratory, IRCCS Istituto Centro San Giovanni di Dio Fatebenefratelli, Brescia, Italy; 4 Nanoscience Laboratory, Department of Physics, University of Trento, Povo (TN) Italy; 5 Department of Electrical and Electronics Engineering, Bahçeşehir University, Istanbul, Turkey; 6 Dipartimento di Biotecnologie e Bioscienze, Università di Milano-Bicocca, Milano, Italy; 7 Department of Electrical and Computer Engineering, Boston University, Boston, Massachusetts 02215, USA

## Abstract

Exosomes, which are membranous nanovesicles, are actively released by cells and have been attributed to roles in cell-cell communication, cancer metastasis, and early disease diagnostics. The small size (30–100 nm) along with low refractive index contrast of exosomes makes direct characterization and phenotypical classification very difficult. In this work we present a method based on Single Particle Interferometric Reflectance Imaging Sensor (SP-IRIS) that allows multiplexed phenotyping and digital counting of various populations of individual exosomes (>50 nm) captured on a microarray-based solid phase chip. We demonstrate these characterization concepts using purified exosomes from a HEK 293 cell culture. As a demonstration of clinical utility, we characterize exosomes directly from human cerebrospinal fluid (hCSF). Our interferometric imaging method could capture, from a very small hCSF volume (20 uL), nanoparticles that have a size compatible with exosomes, using antibodies directed against tetraspanins. With this unprecedented capability, we foresee revolutionary implications in the clinical field with improvements in diagnosis and stratification of patients affected by different disorders.

Exosomes are a class of membranous extracellular vesicles (EV) that originate from inward budding of the endosomal compartment within a cell^1^. The interest of scientists and physicians in EVs has grown dramatically over the past decade in response to the discovery that these vesicles transfer mRNA, miRNA, and protein from the cell of origin to recipient cells^2^, serving a new route for cell-to-cell communication. Presence of exosomes in circulating bodily fluids, including blood^3^, urine^4^ and saliva^5^, suggests that minimally-invasive diagnosis of a number of diseases can be achieved through detection of these vesicles^6^,^7^,^8^,^9^. In particular, EVs are considered valuable for liquid biopsies in cancer diagnosis since they carry molecular and proteomic cargo from their tumour cell of origin^10^. In human CSF, EVs are rich reservoirs of biomarkers for neurological disorders and there is increasing evidence that deregulation of EVs secretion play a pathological role in neurodegenerative diseases such as Alzheimer’s disease (AD) and Frontotemporal dementia (FTD)^11^,^12^,^13^,^14^,^15^.

The limited utility of exosomes in diagnostics is mainly due to difficulties in specifically characterizing them using a scalable phenotyping method. Exosomes have diameters in the range from 30–100 nanometers, i.e., which is too small to be accurately sized by conventional methods such as optical microscopy and flow cytometry (FC) without labels. Alternatively, immunocapturing of exosomes on antibody coated beads^16^ facilitates analysis by FC. However, such indirect detection is not quantitative and measurements are further complicated due to aggregation of exosome-bead complexes. A number of references report on the direct visualization of exosomes with electron microscopy^17^ but this technique is not suitable for large scale application due to its complexity and low-throughput. Western blot is currently used to verify that isolated vesicles are indeed exosomes through characterization of exosomal proteins. However, even the analysis for a single antigen demands large amounts of purified exosomal proteins isolated by extensive and time-consuming (hours to days) procedures. Such requirements limit the throughput and substantially increase the cost for multi-parameter measurements.

The efforts in development of new tools for analysis of exosomes have led to a number of innovative technologies with potential clinical applications. Two recently commercialized nanoparticle detection technologies are typically utilized in characterization of exosomes: Nanoparticle Tracking Analysis (NTA) (NanoSight) and conductivity measurements across a porous membrane (qNano by Izon Science Ltd). NTA is the most commonly used method for determining size distribution and concentration of isolated exosomes in suspension where particle size is calculated based on Brownian motion^18^. In order to overcome the limitations of conventional NTA to determine the cell of origin and to distinguish between different vesicles types (i.e. EVs, lipids and protein aggregates), a short wavelength (405-nm blue-violet) laser and a high sensitivity camera to detect fluorescent particles^19^ are incorporated to the optical system. In this modality, combining NTA with fluorescence measurements, only exosomes labeled with specific fluorescent antibodies are detected, thus allowing their phenotype to be determined. Despite this improvement, challenges persist due to the difficulty in multiplexing and large volume requirements. TRPS measures the size and concentration of a nanoparticle suspension through conductivity changes through a porous membrane^20^, but no information is provided on the nature of the protein expressed on the surface. Both techniques provide valuable information but they cannot identify and simultaneously phenotype exosomes, which is an important limitation, as the actual presence of certain surface proteins would allow the identification of exosomes originating from different cell sources.

There are several emerging new techniques for label-free detection of exosomes. Recently, a real-time, label-free sensing of single exosomes in serum using antibody functionalized micro-toroid optical resonators has been introduced^21^. Although highly sensitive, such high-Q optical resonators have significant challenges of identifying size of captured particles in a complex solution and difficulty of multiplexing.

Another real-time, label-free exosome assay based on surface plasmon resonance (SPR) has been demonstrated^22^. The plasmonic sensor comprises of an array of periodic nanoholes patterned in a metal film. Binding to the sensor surface is monitored through change in optical transmission due to change of refractive index at the sensor surface. The technology offers high sensitivity and enables continuous and real-time monitoring of molecular binding. However, this sensor technology does not provide any information about the size of the captured target to indicate if the signal is due to capture of intact vesicles or soluble proteins.

The study presented in this paper demonstrates a label-free detection platform for routine analysis of exosomes using antibody-based exosome arrays and interferometric digital detection of individual nanoparticles (NPs). The technique enables the fractionation of populations of exosomes that exhibit specific surface proteins allowing better understanding of EV heterogeneity. By comparing the phenotype and size of EVs present in body fluids of healthy donors and patients with different diseases, the platform is expected to accelerate the translation of exosome studies from research into clinics. The technology proposed in this article, based on interferometric imaging, can detect individual nanovesicles on a capture surface in a microarray format and, potentially, assess their size. The principle of Single Particle Interferometric Reflectance Imaging Sensor (SP-IRIS) technology is the enhanced contrast in the scattering signal from nanoparticles that is generated using a layered substrate. The technology has so far mostly been applied to the simultaneous detection of multiple viruses in serum or whole blood^23^. By employing affinity-based capture, size discrimination, and a digital detection scheme to count single virus particles, a robust and sensitive virus/nanoparticle sensing assay was established for targets in complex samples. The SP-IRIS platform has been previously shown to count and size individual viruses from complex solutions at low concentrations down to 5 × 10^3^ PFU/mL in serum^23^. In this work, we demonstrate that the SP-IRIS platform can count and phenotype multiple types of exosomes from a single sample. The contrast of the detected nanovesicles in the SP-IRIS image can be used to estimate the relative size of the nanovesicles, while their spatial location in the array provides information on the exosomes’ phenotype. The novelty of this work is that a single instrument provides the size and multi-phenotype information from the same exosome preparation.

## Materials and Methods

### Materials

Phosphate buffered saline (PBS), Trizma base (Tris), HCl, ethanolamine, ammonium sulphate, and Tween-20 were purchased from Sigma-Aldrich (St. Louis, MO, USA). Copoly(DMA-NAS-MAPS) (MCP-2) was purchased from Lucidant Polymers Inc. (Sunnivale, CA, USA). SP-IRIS printed microarrays were provided by nanoView Diagnostics Inc.

Monoclonal antibodies against the human tetraspanins CD63, CD81, CD9, and CD171 were purchased from Nexgenarrays LLC (Boston, MA). Pure whole molecules IgG from Goat (R&D Biosystems) or Rabbit (Jackson Immunoresearch, West Grove, PA, USA) were used as negative controls.

Artificial cerebrospinal fluid was composed of NaCl (120 mM), NaHCO_3_ (25 mM), KCl (2.5 mM), NaH_2_PO_4_ (1 mM), CaCl_2_ (2.5), MgCl_2_ **⋅** 6 H_2_O (1 mM), Glucose (20 mM) and Human Serum Albumin (0.4 mg/mL), all purchased from Sigma-Aldrich (St. Louis, MO, USA).

### Exosome isolation from human biological samples

HEK 293 (Human Embryonic Kidney 293) cell line were cultured in Minimum Essential Medium (MEM, Life Technologies) supplemented with 10% Fetal Bovine Serum (EuroClone S.p.A.), 1% Penicillin/Streptomycin solution (Carlo Erba Reagents), 2 mM L-glutamine (Carlo Erba Reagents), 1% MEM non-essential Amino Acids solution (Carlo Erba Reagents), at 37 °C in 5% CO_2_/95% air. Cells were grown to complete confluence and incubated in serum-free medium for 72 h. Conditioned media were pooled and centrifuged to obtain exosomes according to a standard protocol^24^. Briefly, conditioned media were collected and spun at 300 × g for 10 min three times. The supernatants were then sequentially centrifuged at 1200 × g for 20 min and again at 10,000 × g for 30 min followed by ultracentrifugation at 110,000 × g for 60 min (T-8100 rotor) (Sorvall). All centrifugations were performed at 4 °C. The 110,000 × g pellet was then resuspended in PBS (5 uL per Petri 100 mm dish) and then serially diluted for NTA and SP-IRIS analyses.

Human cerebrospinal fluid (hCSF) samples were obtained from patients affected by neuropsychiatric disorders, selected from the Fatebenefratelli Biobank. All experiments were performed in accordance with the rules of the local ethycal committee of Fatebenefratelli Hospital (Brescia Italy) that approved the study (Prot. N. 2/92/p/rm; Prot. N. 52/2014; Prot. N. 44/2016).

Informed consent was obtained from all subjects. For Western blot analysis exosomes were isolated from 1.8 mL hCSF by serial centrifugation as described above; for further characterization, the 110,000 x g pellet obtained from 12 mL of hCSF was loaded into a continuous 0.25–2 M sucrose gradient and after 16 h of centrifugation at 200,000 × g (TH-641 rotor) (Sorvall), fractions were collected from the top of the gradient, diluted with PBS and spun at 110,000 × g for 70 min. All centrifugations were performed at 4 °C. The pelleted fractions were analyzed by Western Blot using the TSG101 monoclonal antibody (4A10) (Abcam), the Flotillin-2/ESA antibody (BD Biosciences), the cystatin C polyclonal antibody (Upstate Biotechnology), and monoclonal calnexin antibody (Transduction Laboratories).

### Nanoparticle Tracking Analysis (NTA)

Nano-Sight LM10 instrument (Malvern, Worcestershire, UK) was used to analyze suspensions containing vesicles. The diluted samples were illuminated by a monochromatic laser beam at 532 nm to register a 60 second video taken with a mean frame rate of 30 frames/s. The NTA software (version 3.0, NanoSight) was used to analyze EVs samples, optimized to first identify and then track each particle on a frame-by-frame basis, and its Brownian movement is tracked and measured from frame to frame. Particle size was determined applying the two-dimensional Stokes–Einstein equation based on the velocity of particle movement. From each video, the mean, mode, and median EVs size was used to calculate samples concentration expressed in nanoparticles/mL.

### Coating of microarray silicon chips with copoly-(DMA-NAS-MAPS)

Silicon chips were coated according to the protocol described by M. Cretich *et al.*^25^. Briefly, silicon chips were immersed in a copoly-(DMA-NAS-MAPS)^26^ solution (1% w/v in 0.9 M (NH_4_)_2_SO_4_) for 30 min. The chips were then rinsed with water, dried under nitrogen and cured for 15 min under vacuum at 80 °C.

### Brief description of the SP-IRIS platform

SP-IRIS measurements were carried out using a NVDX10 reader (Nexgenarrays LLC, Boston, MA). The reader automatically acquires interferometric images of the microarray. Nanoparticles bound to the surface of the sensor appear as bright diffraction limited spots that are quantified using the NVDX10 analysis software. NVDX10 software counts nanoparticles captured on the antibody spots within a user defined particle contrast window. For exosome analysis the size window was selected as 1–20% to include particle sizes from 50–200 nm.

### Digital detection of exosomes either from HEK cell line and hCSF using SP-IRIS

Capture antibodies against human CD63, CD81, CD9, CD171 together with negative controls IgG (Goat or Rabbit, pure whole molecules) were arrayed on copoly-(DMA-NAS-MAPS)-coated SP-IRIS patterned silicon chips, with 100 nm oxide layer thickness, using a SciFlexArrayer S5 spotter from Scienion (Berlin, Germany). Antibodies were printed at 1–2 mg/mL in 4 replicates, using PBS as printing buffer; the volume of spotted drops was 265 pL. Printed chips were placed in a humid chamber and incubated overnight at room temperature. Then they were blocked with 50 mM ethanolamine solution in 1 M TRIS/HCl pH 9, for 1 h, washed with pure water and dried under a nitrogen stream. Antibody arrays were first characterized by quantifying the immobilization density of the antibody probes using label-free film thickness measurement using IRIS^27^. IRIS images were taken and analyzed by Nexgenarrays LLC (Boston, MA) QC10 software. The QC software reports the probe density for the immobilized antibodies on the array. According to NanoSight quantifications, vesicles standard preparations were diluted in PBS to a concentration of 6.75E + 10 and 0 particles/mL and then 20 uL of each sample was incubated overnight in static conditions on printed chips. Similarly, 20 uL of hCSF was incubated overnight at room temperature without any dilution. After incubation, chips were washed on a shaker with PBST (PBS with 0.05% Tween-20), PBS and pure water, rinsed in pure water and dried. Label-free SP-IRIS post-incubation scans were acquired and compared to previous corresponding detections using NVDX10 analysis software. The effective vesicle binding to the capture antibodies was obtained subtracting the signals measured after and before sample incubation. Net values from four spot replicates were averaged. Statistical analysis was performed with GraphPad Prism software.

### SEM analysis of SP-IRIS substrates incubated with exosomes

For SEM imaging of captured exosomes the chips were fixed and stained after regular washing procedure but before final drying step. First, the exosomes were fixed with 0.1% gluteraldehyde in PBS for 15 minutes, then rinsed in Millipore water and dried. The chips were then stained in 1% osmium tetroxide in PBS for 30 minutes, then washed three times in Millipore water and dried under pure nitrogen stream. The stained chips were imaged using side scatter detector on a Zeiss Supra 55vp.

### AFM analysis of SP-IRIS substrates incubated with exosomes

AFM measurements were performed on an NT-MDT instrument (SMENA head) working in tapping mode, in air and using a NSG-10 tip (240 kHz resonant frequency, 11.8 N/m force constant). Images of 20 × 20 um were acquired at 1024 points (19.5 nm step) to assure a proper mapping of the EVs. Images acquired at 2048 points showed an increase of nanoparticle size of less than 10%. The software Nova Px 3.4.0 and Gwyddion 2.44 were used for data analysis: for each acquired image, after its planarization, a proper mask was constructed to mark particles higher than 15 nm. The instrument was calibrated with a grating of 19 ± 1 nm height, thus the height accuracy of the nanoparticles mapped in the images is approximately 1 nm.

## Results and Discussion

### Exosomes detection with SP-IRIS, SEM and AFM

**Figure 1:**
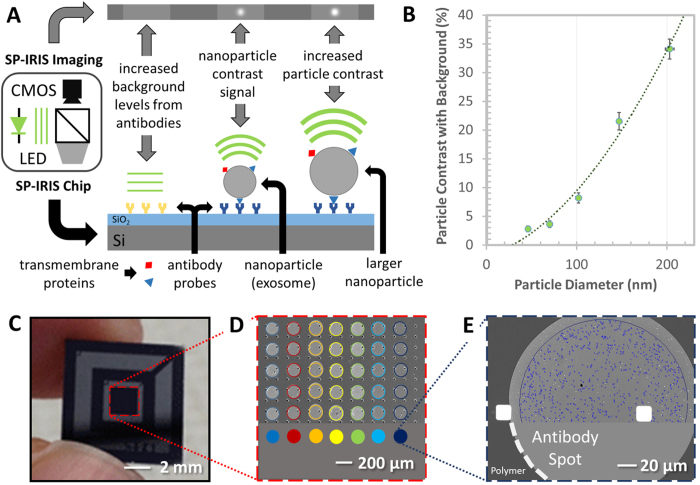
Schematic representation of the SP-IRIS detection process. (**A**) SP-IRIS detection principle, monochromatic LED light illuminates the sensor surface and the interferometriclly enhanced nanoparticle scattering signature is captured on a CMOS camera. (**B**) Demonstrates SP-IRIS signal for polystyrene nanoparticles with a diameter from 50–200 nm which can be used to infer size of captured EVs. (**C**) Image of the SP-IRIS chip. (**D**) Low-magnification interferometric image showing microarray of immobilized capture probes. (**E**) SP-IRIS image of a capture probe. NVDX analysis software recognize capture spot and detects nanoparticles captured.

The lack of high-throughput methods to assess EVs’ phenotype and determine their size and concentration in biological fluids inhibits progress in the validation and clinical use of this class of biomarkers. Standardization and consolidation of methods and protocols that display high feasibility, reliability and reproducibility would speed up the translation of basic scientific findings in clinically relevant applications. In this work we demonstrate the use of an innovative technological platform termed Single Particle Interferometric Reflectance Imaging Sensor (SP-IRIS) for the high-throughput characterization of exosome concentration, relative size distribution and phenotyping in biological fluids^28^. The detection principle for SP-IRIS is based on the enhanced contrast in the scattering signal from particles captured on a silicon substrate with a thin silicon dioxide layer (Fig. 1).

To detect and size nanoparticles, IRIS shines light from visible LED source onto nanoparticles bound to the sensor surface, which consists of a silicon dioxide layer on top of a silicon substrate. Interference of light reflected from the sensor surface is modified by the presence of particles producing a distinct signal that correlates to the size of the particle (Fig. 1A,B). The detection of low-index dielectric particles with diameters of 50 nm to 200 nm is shown in Fig. 1B^28^ demonstrating that the SP-IRIS signal correlates with the size of the particle.

**Figure 2:**
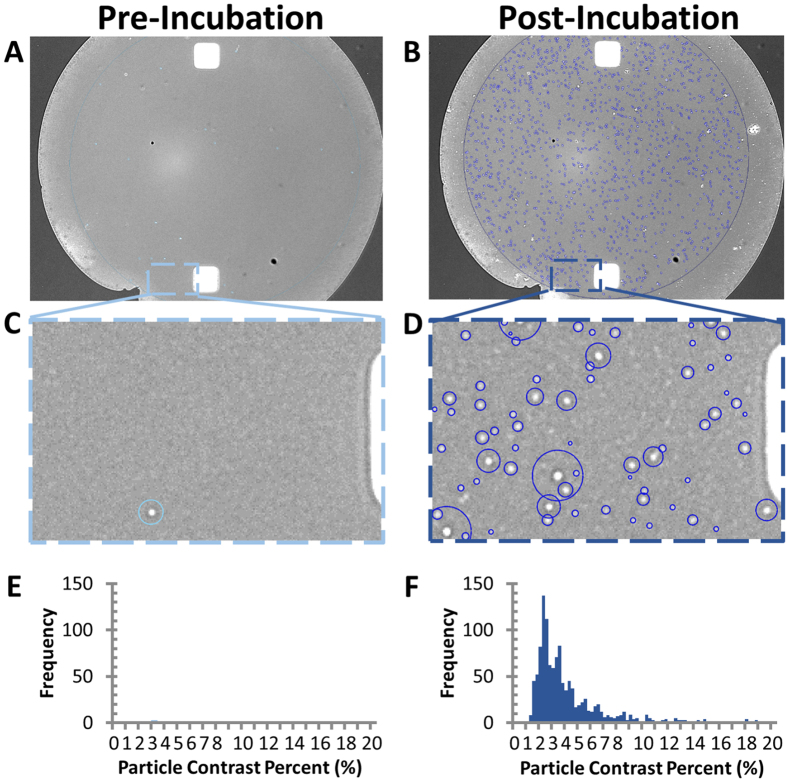
Exosome capture, digital counting, and relative sizing. (**A,B**) Anti-CD81 capture probe image acquired before and after incubation with purified HEK293 cells derived exosomes. (**C,D**) Zoom-box of particles detected pre- and post-incubation. (**E–F**) Particle contrast histogram pre- and post-incubation.

In this work, EVs are captured on the surface of silicon chips through antibodies targeting exosomal markers. The chip’s glass surface is similar to that of a standard microarray slide and is coated with a functional polymer, copoly-(DMA-NAS-MAPS)^26^, on which antibodies, specific for exosome surface antigens, are spotted in a microarray format. In Fig. 1 the analysis steps are shown. The process can be divided into three phases: antibody spotting, exosome capturing and image analysis (Fig. 1C–E). To demonstrate exosome capture and digital counting, exosomes purified by ultracentrifugation from HEK 293 cell line cultures and characterized by NanoSight (Supporting Information, Figure SI1) were captured on anti-CD81 capture antibody spots. Dots of different contrast were clearly distinguished from the background after the chips were incubated with the HEK 293 exosome sample. EVs that, upon incubation, became visible on the spot surface, as shown in Fig. 2, were individually counted by an automated software and surrounded by circles which correspond to an area with contrast higher than the background and represent countable unlabeled individual exosomes (Fig. 2C–F). In the analysis phase particle counts are plotted as a function of their contrast and the distribution is shown in Fig. 2E,F.

**Figure 3:**
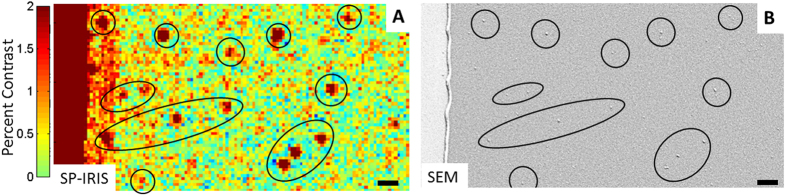
Nanoparticle capture validation with SEM. (**A**) SP-IRIS image of exosomes being captured by anti-CD81 antibody. (**B**) Exosomes visualized by SEM of the same field-of-view for comparison. Scale bar is 1 micron.

To demonstrate that high contrast dots correspond to exosomes, scanning electron microscopy (SEM) and atomic force microscopy (AFM) images of the chip were taken and aligned with SP-IRIS images collected in dry conditions at end-point. In Fig. 3, a portion of capture antibody spot against CD81, after incubation with exosomes purified from HEK cell line (5.27E + 10 particles/mL) and analyzed in SP-IRIS, is compared to the same area observed in SEM. Nanoparticles (NPs) detected with SP-IRIS (Fig. 3A) are circled and correlate with the circles on the SEM image (Fig. 3B). SP-IRIS and SEM showed perfect correspondence for particles larger than 65 nm, while smaller particles are detectable only with electron microscopy, which is consistent with detection limits of both analytical techniques. The particle sizes detected by SP-IRIS ranged between 65–85 nm as measured using the SEM image. No particles were detected with either SP-IRIS or SEM on CD81 antibody spot incubated with exosome depleted HEK293 culture media (Supporting Information, Figure SI2).

**Figure 4:**
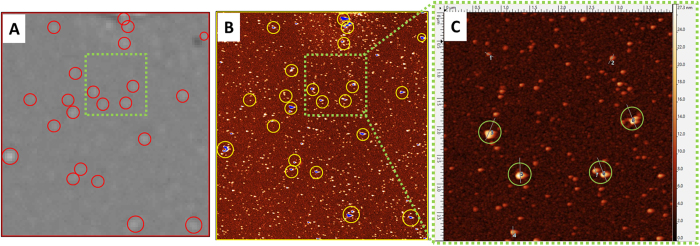
Exosomes purified from HEK cell line, captured with anti- CD81 antibody on silicon chip and detected by SP-IRIS (**A**) and AFM (**B,C**). (**A**) SP-IRIS Image of the anti-CD81 spot incubated with a suspension of exosomes (6.75E + 10 exosomes/mL), the red circles highlight the countable nanoparticles. (**B**) AFM image of the same spot area: the blue dots identify particles larger than 15 nm; the yellow circles show in image (**B**) perfectly match the particles detected by SP-IRIS in image (**A**). (**C**) Zoom in area of the anti-CD81 spot shown in the green frame highlights the particles larger than 15 nm in height detected by AFM and SP-IRIS. Particles smaller than 15–10 nm are detectable only with AFM as they are below SP-IRIS detection limit.

In Fig. 4, a part of anti-CD81 spot is analyzed by SP-IRIS and aligned with an AFM image of the same area. SP-IRIS images were acquired before (Supporting Information, Figure SI3) and after (Fig. 4A) incubation with exosomes purified from HEK cell line. Red circles in Fig. 4A correspond to vesicles detected after sample incubation. Figure 4B shows the same spot area imaged with AFM.

In a number of publications^29^,^30^, the height of exosomes measured by AFM is as low as 10–20 nm. The size of vesicles on images performed on dry chips (nanoparticles are surrounded by air) may be an underestimation of the real size due to the drying process. Particles with a height profile in excess of 15 nm in the AFM image are considered exosomes and lateral dimension was measured to be between 80–140 nm + /− 20 nm. In the image of Fig. 4B the particles higher than 15 nm, marked in blue coincide with the spots of higher contrast, surrounded by yellow circles, detected by SP-IRIS (red circles). The close up of the area in the green square, shown in Fig. 4C provides a higher resolution AFM image of the surface. SP-IRIS detects vesicles that appear to be higher than 15 nm (Supporting Information SI4), whereas smaller particles go undetected in SP-IRIS. No particles were detected on CD81 antibody spot incubated with exosome depleted HEK293 culture media (Supporting Information, Figure SI5).

### SP-IRIS phenotyping of exosomes purified from HEK cell

**Figure 5:**
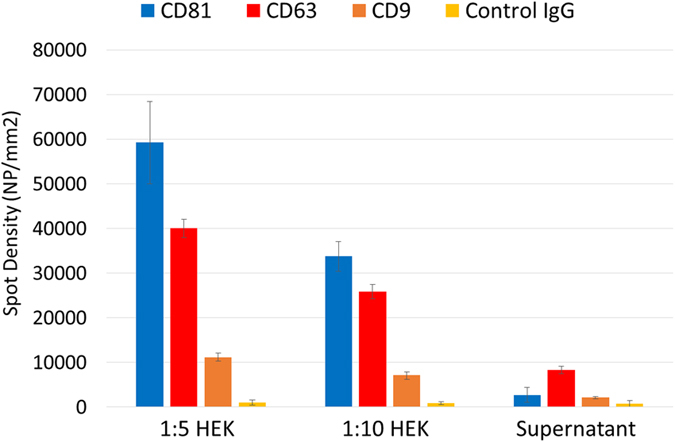
Exosome Phenotyping. Exosomes, isolated from HEK fibroblast cells and EV depleted supernatant, captured with antibodies against CD81, CD63, CD9 and IgG negative control and detected by SP-IRIS.

Three capture antibodies against the exosomal biomarkers CD81, CD63 and CD9 were arrayed on the same chip together with a negative control IgG in order to evaluate the vesicle tetraspanin expression profile (Supporting Information, Figure SI6). Exosomes purified from HEK cell line and the EV depleted supernatant (SN, as negative control) were analyzed with NTA (Supporting Information, Figure SI1) to determine the particle concentration in each sample. Exosomes were diluted in PBST at 5.27 and 2.64E + 10 particles/mL. In Fig. 5, the histogram reports the number of vesicles, per square millimeter, detected by SP-IRIS. The results indicate higher expression of CD81 tetraspanin compared to CD63 and CD9. As expected, the EVs depleted SN showed no binding to CD81 and CD9, but low binding to CD63. No significant particles were counted on control IgG spots. The relative size of the exosomes captured by CD81, CD9, and CD63 was measured using SP-IRIS through particle contrast quantification (Supporting Information, Figure SI6). Based on polystyrene nanoparticle response (Fig. 1B), the estimated mean diameter of the exosomes is 55–65 nm. Also much larger particles were observed whose diameter is comparable to that measured by AFM and SEM.

By establishing a dilution curve with exosome standards the user can determine the EVs concentration for different surface phenotype using a single microarray. The exosomes detected by the tetraspanin probes showed similar size distribution as inferred by the measured contrast distribution of the particle in the SP-IRIS image (Supporting Information, Figure SI6).

### Dilution curves of exosomes purified from HEK cell line and assay limit of detection

Exosomes purified from HEK cell line and EV depleted supernatant (SN, negative control) were characterized by NTA (Supporting Information, Figure SI1) in order to evaluate the particle content in each sample. Several dilutions ranging from 6.8E + 10 to 9E + 09 particles/mL were incubated on silicon chips where capture antibodies against CD81 and CD63 as well as Rabbit IgG (R IgG, as negative control) were arrayed. As NTA was unable to detect any particle in the SN, this sample was used as control.

**Figure 6:**
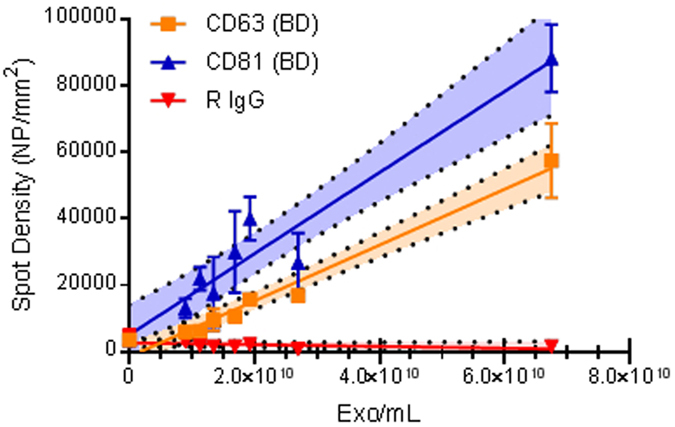
Dilution curve of exosomes purified from HEK cell line and detected with SP-IRIS. A good correlation can be observed for both capture antibodies CD63 (yellow line, R^2^ 0.97) and CD81 (blue line, R^2^ 0.93); no particles were detected on the negative control R IgG (red line). The bright area around the regression line corresponds to the 95% confidence interval. Based on line equations, limits of detection (LOD) were calculated, resulting in 5.07E + 09 particles/mL for CD63 antibody and 3.94E + 09 particles/mL for CD81 antibody.

Figure 6 shows the dose-response curves for particles counted on each antibody by SP-IRIS. The regression area around each line corresponds to 95% confidence interval of the data. As expected, no particles were detected on the negative control R IgG. A linear correlation was observed between exosome concentration and particle counts with excellent correlation coefficients (R^2^) for both capture antibodies: CD81 (0.93) and CD63 (0.97). By using the equations of the extrapolated lines, detection limits were calculated for both tetraspanins: 3.94E + 09 and 5.07E + 09 particles/mL, respectively for CD81 and CD63.

Detection limit comparison between SP-IRIS target specific capture and NTA warrants further investigation. NTA measures non-specifically all particles in a solution, while SP-IRIS is only measuring the subpopulation based on the capture antibody target on the sensor’s surface. It has been reported in the literature that exosome preparations by pelleting through ultracentrifugation contain a heterogeneous population of exosomes with different proteomic and molecular composition^31^. Further experiments are required using exosome standard with known surface marker composition to allow direct comparison of analytical sensitivity between NTA and SP-IRIS.

### SP-IRIS detection and quantification of exosomes in human cerebrospinal fluid (hCSF)

**Figure 7:**
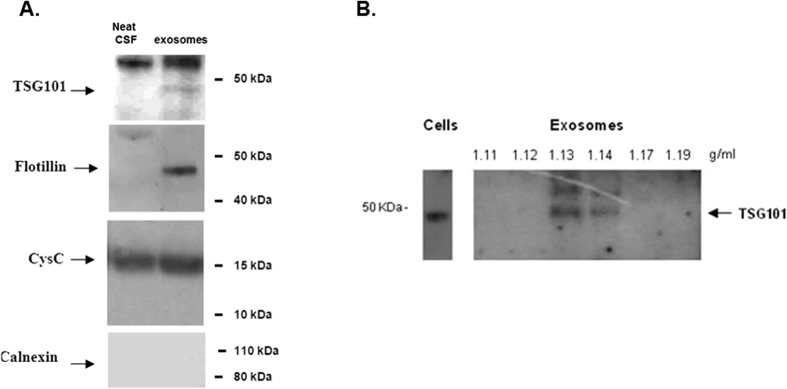
(**A**) Western blot analysis of exosomes isolated from 1.8 ml of hCSF and neat hCSF: the exosomal markers TSG101 and flotillin marked the exosomes fraction and not neat CSF, cystatin C marked both fractions and calnexin was not detected. (**B**) Sucrose gradient fractions of exosomal preparations from hCSF were immunoblotted with the TSG101 monoclonal antibody. TSG101 marked the exosomes-positive fractions (corresponding to 1.13 and 1.14 g/mL sucrose). HEK cells lysate was used as positive control. Western blot gels have been cropped to show only the relevant protein bands. Complete figures can be found as Supplementary, Figure SI 7.

As exosomes are vesicles released by every kind of cell, they have been found in several human biological fluids, such as urine, saliva, blood as well as cerebrospinal fluid (CSF)^32^. Determining the concentration and size of exosomes in human CSF (hCSF) is not trivial, because the available sample volume is usually too small to allow analysis either with NTA or ELISA. In this work, hCSF has been used to demonstrate the potential of clinical utility of the technique. A conventional way to characterize exosomes in hCSF requires serial centrifugations in a density gradient followed by a characterization by Western blot. We first characterized exosomes in hCSF by a conventional approach and the results are shown in Fig. 7A,B. The exosomal proteins TSG101 and flotillin were present in the exosomal fractions and not in CSF depleted of exosomes (Neat CSF) while cystatin C (CysC), a soluble protein which is also secreted in association with exosomes^11^, was present in both fractions. Conversely, calnexin, an endoplasmic reticulum residential protein, was absent from both fractions indicating the absence of cell fragments contamination (Fig. 7A). Furthermore TSG101 was used as a marker to identify the fractions containing exosomes in a sucrose gradient. The results of Fig. 7B confirm that the 1.13 and 1.14 g/mL sucrose fractions are enriched in exosomes.

**Figure 8:**
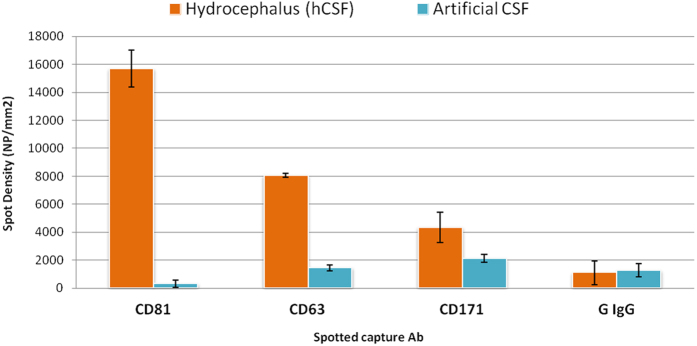
SP-IRIS label-free assay on human hydrocephalus CSF sample and artificial CSF. The expression of the typical exosomal biomarkers CD81 CD63 as well as the neural adhesion protein CD171 is significantly different than in the artificial CSF, negative control. An additional negative control, a non correlated IgG, shows a low level of non-specific binding. Each multiplexed chip was run in duplicate independent replicate tests.

While the analysis reported above, required 1.8 mL of hCSF, only 20 uL of sample were required by SP-IRIS. The 20 uL of hCSF were incubated over-night, without any dilution, on chips multiplexed with antibodies against tetraspanins (CD81 and CD63) as exosome markers and CD171, a cell adhesion molecule highly expressed by neuronal cells commonly used to capture exosomes of neural sources from blood^33^, together with a control IgG antibody. As negative control, an artificial CSF was used. SP-IRIS images after hCSF incubation were compared with pre-scan acquisitions and the data analyzed by NVDX10 Analysis Software. Figure 8 reports the results of the SP-IRIS label-free assay on hCSF. Signals on CD81 and CD63 are different, indicating that not all tetraspanins are expressed equally. Also CD171 showed higher binding in hCSF than negative control sample.

Considering the average particle densities on CD81 and using the calibration curves previously obtained for each capture antibody (Fig. 6), an average concentration of about 2E + 10 particles/mL in hCSF was extrapolated.

## Conclusions

The characterization and quantification of circulating as well as cell culture-derived exosomes based on expression of specific exosomal biomarkers (e.g. tetraspanins, annexins and heat shock proteins) is currently performed by running size and concentration measurements on vesicles and immunoproteomic analyses in parallel and then correlating the results. Size and concentration measurements are performed with Electron Microscopy, Nanoparticle Tracking Analysis (NTA) (NanoSight) or qNano (Izon Science Ltd). However, these techniques can only be used to characterize pure EVs as they do not discriminate nanoparticles of different origin, for instance exosomes from protein aggregates or lipids of the same size. Even though they can objectively define the EVs size range and concentration, they are restricted in their ability to simultaneously phenotype them. Therefore, phenotyping is done in parallel using immunoproteomic techniques like immunoblotting or by a novel, high resolution, flow cytometric analysis where exosomes are captured on antibody-coated latex beads^16^. This latter type of analysis allows the simultaneous evaluation of multiple parameters on single exosomes by a multicolor labeling strategy. However, conventional flow cytometry and Western Blotting demand large amounts of proteins isolated by extensive and time-consuming standard isolation procedures.

Therefore, direct counting, sizing and phenotyping of EVs will streamline both analysis and characterization. SP-IRIS platform can perform phenotyping and, potentially, sizing at the single exosome level without the need to correlate two separate measurements, which could be inaccurate, especially when done on non-purified samples because exosomal marker targets could be in soluble form not associated with nanoparticles. SP-IRIS can enumerate, estimate particle size, and phenotype exosomes from purified samples from cell culture, or directly from just 20 uL of a clinical sample such as hCSF. A detection limit of 3.94E + 09 particles/mL was achieved for purified nanoparticles from HEK 293 cell lines, as quantified using NTA. Exosome quantification by NTA is non-specific and therefore concentration of nanoparticles measured is an overestimate of the exosome concentration. Therefore, further experiments need to be done to understand the detection limits differences. In addition, we demonstrated that SP-IRIS can potentially phenotype exosomes directly from hCSF, employing a very small sample volume.

## Additional Information

**How to cite this article**: Daaboul, G. G. *et al.* Digital Detection of Exosomes by Interferometric Imaging. *Sci. Rep.* **6**, 37246; doi: 10.1038/srep37246 (2016).

**Publisher’s note**: Springer Nature remains neutral with regard to jurisdictional claims in published maps and institutional affiliations.

## Supplementary Material

Supplementary Information
