# Supplementary material for: Digital Detection of Exosomes by Interferometric Imaging
Source: Sci Rep. 2016 Nov 17;6:37246. doi: 10.1038/srep37246 (PMC5112555; doi:10.1038/srep37246)

**DIGITAL DETECTION OF EXOSOMES BY INTERFEROMETRIC IMAGING**

**George G. Daaboul1*§, Paola Gagni2§, Luisa Benussi3, Paolo Bettotti4, Miriam Ciani3, Marina Cretich2, David Freedman1, Roberta Ghidoni3, Ayca Yalcin Ozkumur5, Chiara Piotto4, Davide Prosperi6, Benedetta Santini6, M. Selim Ünlü7, Marcella Chiari2***

**1) Nexgen Arrays, Boston, Massachusetts 02215, USA**

**2) Consiglio Nazionale delle Ricerche, Istituto di Chimica del Riconoscimento Molecolare (ICRM), Milano, Italy**

**3) Molecular Markers Laboratory, IRCCS Istituto Centro San Giovanni di Dio Fatebenefratelli, Brescia, Italy**

**4) Nanoscience Laboratory, Department of Physics, University of Trento, Povo (TN)**

**5) Department of Electrical and Electronics Engineering, Bahçeşehir University, Istanbul, Turkey**

**6) Dipartimento di Biotecnologie e Bioscienze, Università di Milano-Bicocca, Milano, Italy**

**7) Department of Electrical and Computer Engineering, Boston University, Boston,**

**Massachusetts 02215, USA**

***: corresponding authors**

**Correspondence to marcella.chiari@icrm.cnr.it**

**§: contributed equally**

**Supporting Information**

**Figure SI1**. Example of typical size distribution and nanoparticle tracking analysis (NTA) counting of exosomes purified from HEK cell line. For NTA method the sample preparation is minimal: it has to be at the suitable diluition for the instrument. If it is necessary the sample could be diluited with sterile water after checking the absence of contaminating nanoparticles into the buffer. After capturing, videos the software processes the data giving firstly the mean, the mode and standard deviation of the exosomes size distribution and then their concentration per mL. In the example, exosomes purified from HEK cell line were diluted 1:500 in pure water, providing a concentration of 5.27E+08 particles/mL and a dimension distribution of hydrated vesicles with 118.6 nm peak.


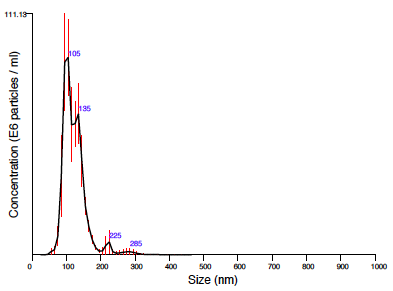

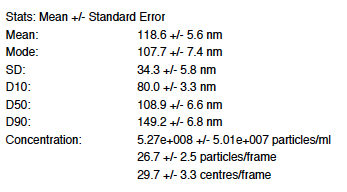


**Figure SI2**. Comparison of SP-IRIS and SEM image for CD81 antibody spot incubated in exosomes depleted cell culture media from HEK293 cells. SP-IRIS and SEM image show no particle binding. Scale bar is 1 micron.

**
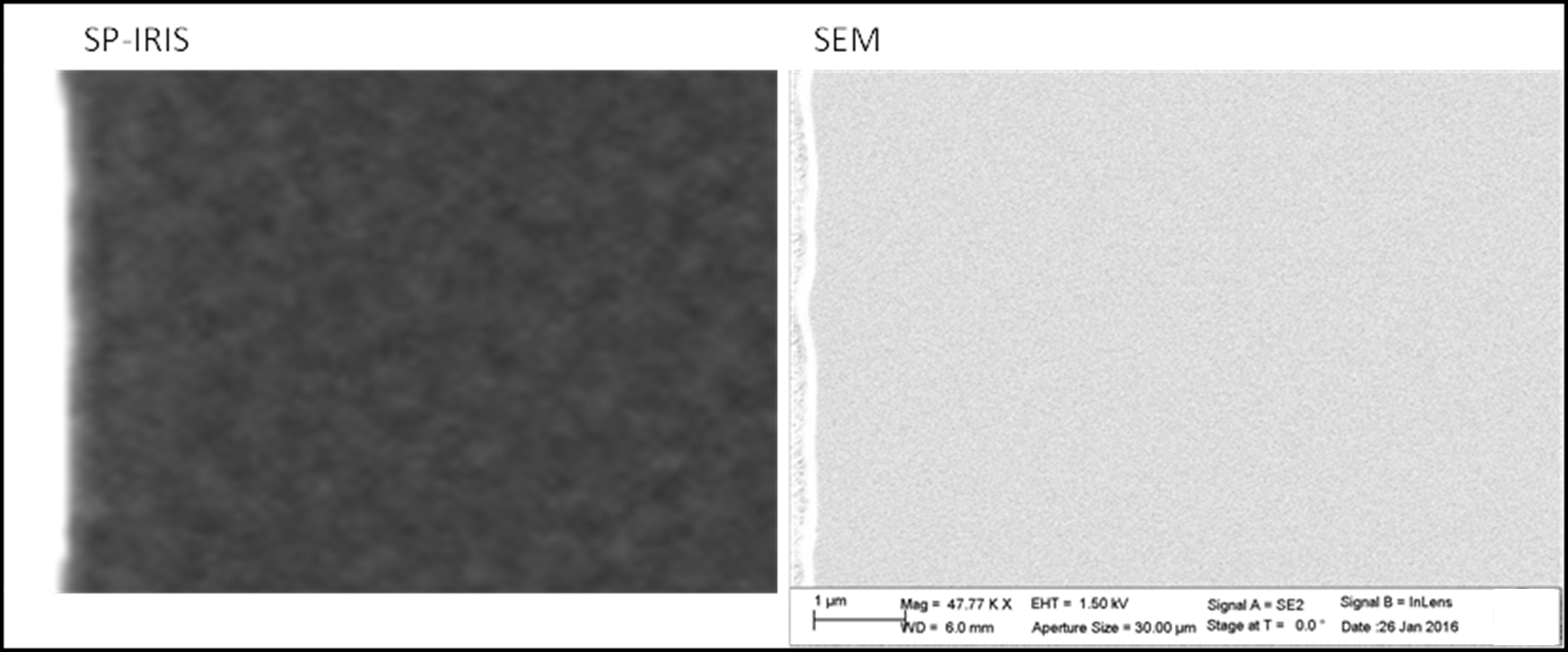
**

**Figure SI3**. SP-IRIS image of anti-CD81 capture antibody before any sample incubation. No particles are detected on the plain spotted antibody. Green dots square the area further magnified with AFM after sample incubation (Fugure 4C).


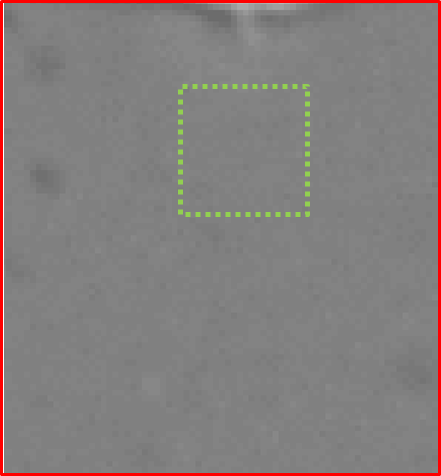


**Figure SI4**. Vesicles height profile measured with AFM of exosomes detected in Figure 4C, highlighted with green circles.


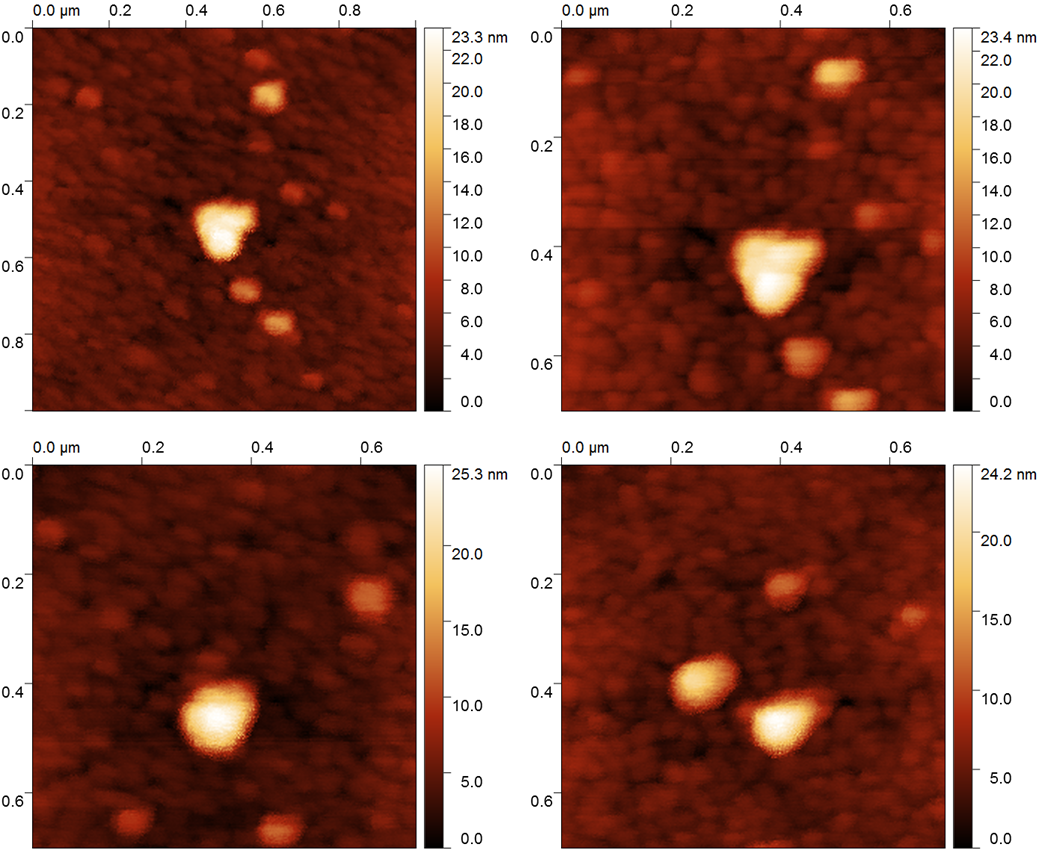


**Figure SI5**. Comparison of SP-IRIS and AFM image for CD81 antibody spot incubated with exosomes depleted cell culture media from HEK293 cells. SP-IRIS and AFM image show no particle binding.


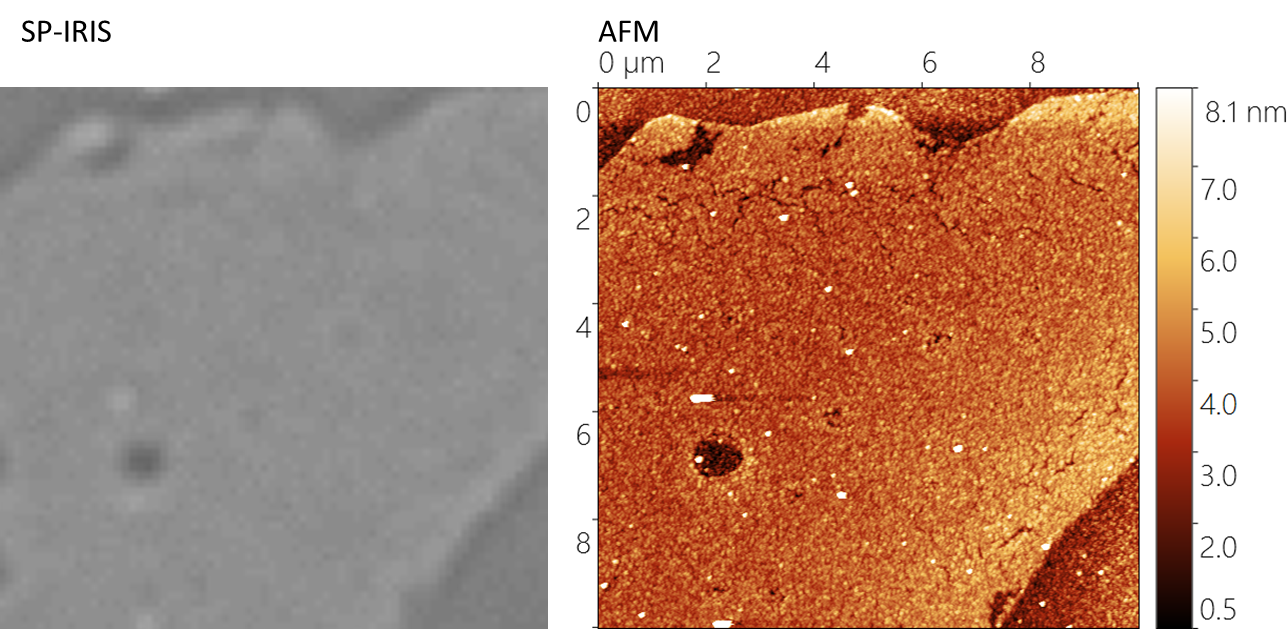


**Figure SI6**. Contrast distribution of particles of HEK exosomes purified by ultracentrifugation (A,B,C) and EVs depleted supernatant (D,E,F) incubated on SP-IRIS chip with anti-CD63 (A,D), anti-CD81 (B,E) and Goat IgG negative control (C,F) spotted on the surface of the chip. The light blue bars correspond to counts on spotted capture antibodies, while the dark blue bars represent the effective particle counts in terms of difference between after and pre-sample incubation. HEK derived exosomes bound to CD63 and CD81 antibody and showed similar size distribution. EV depleted supernatant from HEK cell line showed some residual amount of CD63 positive exosomes. Negative control showed negligible binding for both exosome and supernatant samples.

**Figure SI7**. Western blot analysis (full blots) of A. exosomes isolated from 1.8 ml of hCSF and neat hCSF; B. Full blot of sucrose gradient fractions of exosomal preparations from hCSF.


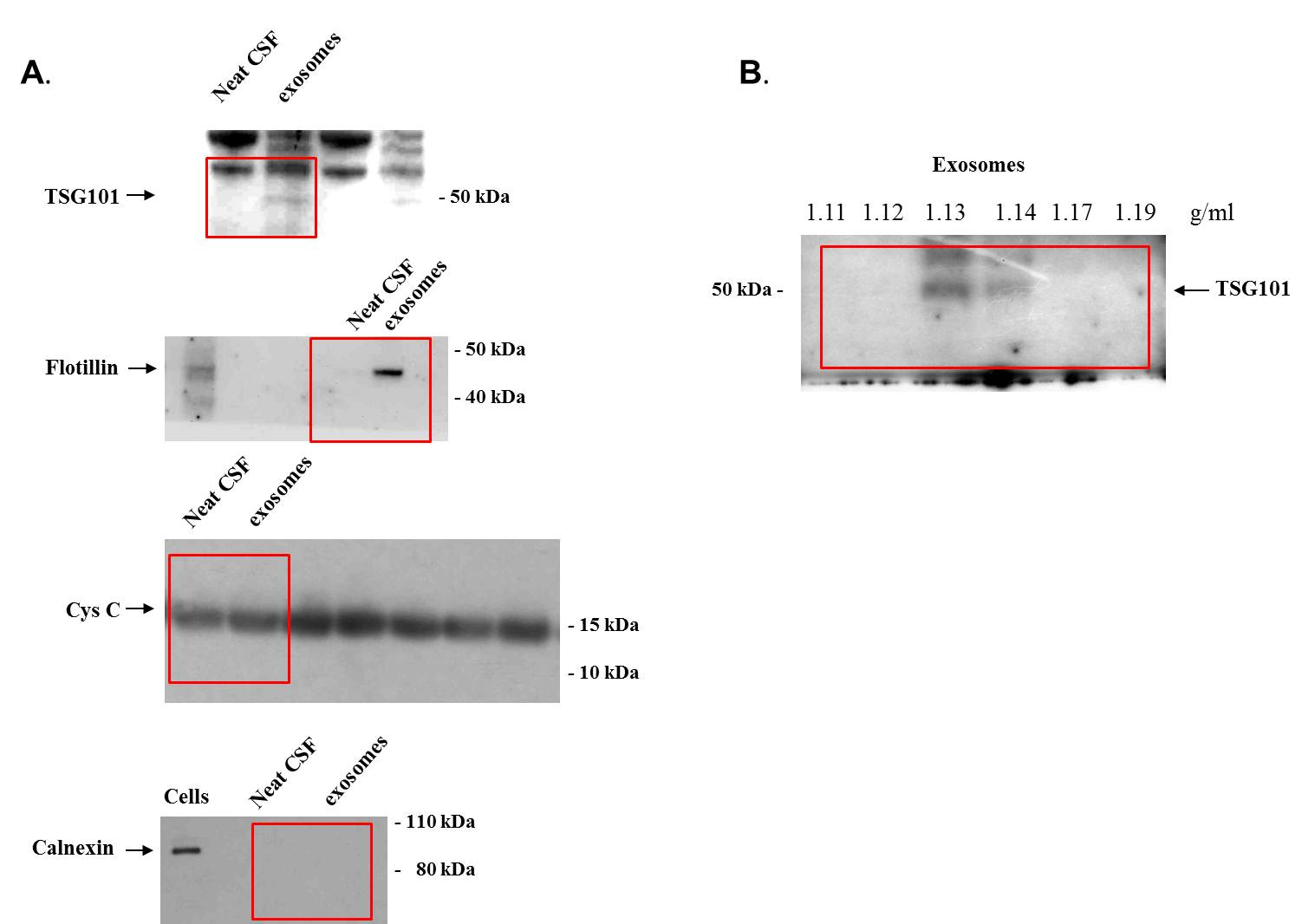

Supplement: Supplementary Information [file srep37246-s1.doc]
